# Wie geht es dem akademischen Mittelbau? Eine Umfrage

**DOI:** 10.1007/s00287-022-01479-8

**Published:** 2022-08-18

**Authors:** Mario Gleirscher, Kerstin Lenk, Jens-Martin Loebel, Tom Petersen, Johannes Wagner

**Affiliations:** 1 Universität Bremen, Bremen, Deutschland; 2 TU Graz, Graz, Österreich; 3 Hochschule Magdeburg-Stendal, Magdeburg, Deutschland; 4 Universität Hamburg, Hamburg, Deutschland; 5 Beirat des Wissenschaftlichen Nachwuchses der Gesellschaft für Informatik (GI WiN), Bonn, Deutschland

## Abstract

Zum sogenannten Mittelbau zählen Doktoranden und Doktorandinnen, Postdocs, Nachwuchsgruppenleiter und -leiterinnen, Junior- und Tenure-Track-Professoren und -Professorinnen. Insbesondere Promovierende sowie Postdoktoranden und Postdoktorandinnen sind, noch mehr als früher, von den komplexen strukturellen und finanziellen Problematiken des Wissenschafts- und Lehrbetriebs betroffen, und das in vielerlei Hinsicht über Fächer hinweg. Der Flaschenhals auf dem Weg zur Professur oder einer anderweitig verstetigten Forschungs- oder Lehrstelle führt in der akademischen Karriere zu prekären Beschäftigungsverhältnissen. Die schwierige Vereinbarkeit von Familie und akademischer Karriere erzeugt eine zusätzliche Benachteiligung, insbesondere von Wissenschaftlerinnen. Mangelnde Qualitätssicherung sowie fehlende zuverlässige und vertrauenswürdige Prozesse erschweren die Aufdeckung und Aufarbeitung von Konflikten während der Promotionsphase und der Zeit des Postdoktorats. Der Beirat des wissenschaftlichen Nachwuchses (GI-WiN) der Gesellschaft für Informatik e. V. (GI) fordert und empfiehlt in einem Positionspapier (10.1007/s00287-020-01250-x) mehrere Maßnahmen zur Stabilisierung von Karriere- und Beschäftigungssituation des Mittelbaus. Der vorliegende Artikel fasst die Ergebnisse einer Umfrage zur Ergänzung und empirischen Untermauerung dieser Empfehlungen zusammen. Die vorliegende Umfrage wurde mit dem Anspruch erhoben, Daten aus allen Fachbereichen – nicht nur der Informatik – zu sammeln. In den Ergebnissen zeigte sich, dass diesen vielfältigen Herausforderungen begegnet werden muss, die in Nicht-MINT-Fächern und MINT-Fächern ähnlich empfunden werden. Die Schaffung von unbefristeten Stellen für die Phase nach der Promotion wurde im Rahmen der Umfrage als besonders wünschenswert angesehen. Vorgeschlagen wurde auch die Abschaffung von Lehrstühlen und die Einführung einer Departmentstruktur. Des Weiteren wurde die Trennung von Begutachtung und Betreuung vor allem bei der Promotion angeregt. Die letzten 2 Jahre im Zeichen der COVID-19-Pandemie waren für einen großen Teil der Betroffenen von fehlendem fachlichen Austausch und sowohl beruflichen als auch privaten Zusatzbelastungen geprägt. Diese Ergebnisse können über die genannten Fachbereiche hinaus eine Entscheidungsgrundlage für eine gerechtere Wissenschaftspolitik liefern und bessere Arbeits- und Karrierebedingungen für den wissenschaftlichen Nachwuchs erwirken.

## Einleitung

Der Begriff des wissenschaftlichen Nachwuchses umfasst Doktoranden und Doktorandinnen, Postdocs, Nachwuchsgruppenleiter und -leiterinnen sowie Junior- und Tenure-Track-Professoren und -Professorinnen, die an Hochschulen und außeruniversitären Forschungseinrichtungen beschäftigt sind und eine Professur oder eine wissenschaftliche Leitungsposition anstreben [1].

Nicht erst seit der im letzten Jahr auf Twitter entbrannten Debatte um die Arbeitsbedingungen und Karriereaussichten unter dem Hashtag #IchBinHanna ist bekannt, dass Angehörige des wissenschaftlichen Nachwuchses oftmals strukturellen und finanziellen Problemen ausgesetzt sind. Unsichere Karriereperspektiven, eine unzureichende Vereinbarkeit von Familie und Beruf und Mehrfachbelastung durch Lehr‑, Forschungs- und administrative Aufgaben sind nur einige der bekannten Herausforderungen [1].

Im Vorfeld dieses Artikels hat der Beirat des wissenschaftlichen Nachwuchses der Gesellschaft für Informatik e.V. (GI-WiN) bereits Empfehlungen zur Verbesserung der Lage in einem Positionspapier [1] zusammengetragen. Dazu zählen die Verbesserung der Betreuung während der Promotionsphase und die Schaffung weiterer Karrierewege. Dies schließt zusätzliche Tenure-Track-Stellen und Lebenszeitprofessuren sowie eine Reform der wissenschaftlichen Leistungsbewertung, der Forschungsförderung und der Hochschulfinanzierung mit ein. Zudem fordert auch der GI-WiN eine Entfristungsoffensive zur Stärkung des Mittelbaus mit Umgestaltung des Wissenschaftszeitvertragsgesetz (WissZeitVG), einhergehend mit der Etablierung von Alternativen zur Professur und Stärkung der Hochschulen für angewandte Wissenschaften. Abschließend macht sich das Positionspapier für die Verbesserung der Betreuungsverhältnisse durch Trennung von Betreuung und Begutachtung und eine externe, unabhängige Begutachtung stark.

Nachfolgend soll nun die Lage des wissenschaftlichen Mittelbaus basierend auf den Ergebnissen einer durchgeführten Umfrage empirisch untersucht werden. Dazu wird im nächsten Abschnitt die angewandte Methodik dargeboten. Anschließend werden die demografischen Daten zu den Onlinebefragten beschrieben. In den darauffolgenden Abschnitten werden die Herausforderungen und die Bewertung von Lösungsvorschlägen aus dem gesammelten Datenmaterial herauskristallisiert. Dabei wird auch auf den Einfluss der COVID-19-Pandemie auf den wissenschaftlichen Mittelbau eingegangen. Der Artikel schließt mit einer Diskussion zum Thema, in der die Ergebnisse in Bezug auf bekannte Ergebnisse eingeordnet werden.

## Methodik

Die Umfrage enthält eine Sektion zu demografischen Fragen sowie weitere Sektionen mit thematisch zusammengehörenden Gruppen von geschlossenen und offenen Fragen. Für den Onlinefragebogen war eine 20-minütige Ausfüllzeit angesetzt. Zur Erstellung und Durchführung haben wir das SoSci-Survey-Werkzeug (https://www.soscisurvey.de/) verwendet, zur Auswertung der Umfrageergebnisse sowie zur Anfertigung der Grafiken GNU R (u. a. mit dem Likert-Paket).

### Zur Verteilung des Fragebogens

Die Umfrage wurde auf mehreren Kanälen im Zeitraum von Juni 2021 bis Februar 2022 jeweils ein- bis dreimal beworben. Die Bitte zur Teilnahme an der Befragung wurde wie folgt verteilt: Neben Kanälen des GI-WiN-Beirats (Webseite, Twitter-Kanal, LinkedIn, Facebook, XING, Teilnehmer WiN-Workshop) und der GI (GI-Radar) wurden diverse Mailverteiler deutscher Universitäten, übergreifende Netzwerke (Netzwerk für Gute Arbeit in der Wissenschaft, Thesis e. V., Gewerkschaft für Erziehung und Wissenschaft) und weitere Kanäle (Veröffentlichung in Forschung und Lehre des Deutschen Hochschulverbandes, Universitäts-Newsletter) kontaktiert und zur Verbreitung genutzt. Unser Antrag für das Social Science (SoSci) Panel (https://www.soscipanel.de) und unsere Anfragen an das Panel für den Bundesbericht Wissenschaftlicher Nachwuchs [2] sowie unsere Bitten an die Junge Akademie und einige andere Organisationen wurden abgelehnt bzw. blieben unbeantwortet.

### Einige Eckdaten

Wir schätzen für die Populationsgröße P = 300.000 (Grobschätzung aller Promovierenden und wissenschaftlich Angestellten fächerübergreifend, nach BuWIN Bericht 2021 [2], Tab. B14) und gehen davon aus, dass wir mittels Werbung etwa E = 5300 Personen erreichen konnten (Grobschätzung per Verteilungstabelle). Somit ergibt sich eine Abdeckung A = 5300/300.000 = 2 %. Bei *N* = 379 Datenpunkten ergibt sich damit die Rücklaufquote R = 379/5300 = 7 %.

### Zu den Analysen und Grafiken

Neben einer Gesamtschau aller Daten vergleichen wir im Folgenden die Antworten nach gruppierten Fachdisziplinen. Wir unterscheiden insbesondere *MINT von Nicht-MINT*, um etwaige Unterschiede zwischen zwei üblicherweise als getrennt betrachteten Wissenschaftsbereichen zu identifizieren. Alle Grafiken spezifizieren die Anzahl *N* der jeweils verwendeten Datenpunkte. Blaue Balken in einigen der Balkendiagramme mit Ordinalskalen kennzeichnen den jeweiligen Median.

## Studienpopulation

Im Rahmen der Umfrage konnten 379 vollständige Ergebnisse gesammelt werden. Die Nutzung vielfältiger Kanäle führte zu einer diversen Verteilung der Teilnehmenden in Bezug auf verschiedene Merkmale.

**Figure 1:**
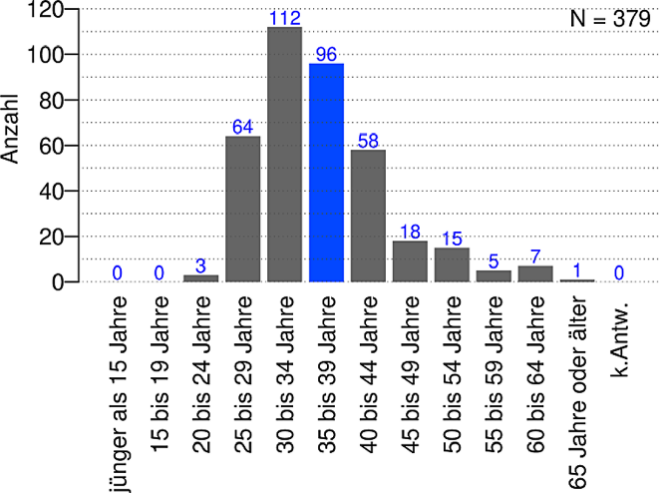

Es nahmen etwa gleich viele weibliche und männliche Personen teil (175 weiblich, 189 männlich). Fünfzehn weitere Personen ordnen sich einem anderen Geschlecht zu oder machten keine Angabe. Der Großteil der Befragten ist zwischen 25 und 44 Jahre alt mit einem Schwerpunkt im Bereich von 30 bis 34 Jahren (Abb. 1). Der überwiegende Anteil der Befragten arbeitet derzeit in Deutschland (367 in Deutschland, 11 andere Angaben und 1 ungültige Antwort).

Bezogen auf das Fachgebiet der Befragten ergibt sich eine breite Streuung. Aufgrund der kontaktierten Kanäle stammt ein großer Teil der Befragten aus dem Bereich der Informatik (40 %). Aber auch Personen aus den Gesellschafts- und Sozialwissenschaften (27 %), Wirtschaftswissenschaften (11 %), Sprach- und Kulturwissenschaften (11 %) und der Mathematik und den Naturwissenschaften (8 %) sind vertreten.

Der überwiegende Teil der Befragten strebt eine Promotion an (45 %) oder hat diese bereits abgeschlossen und arbeitet derzeit als Postdoc (38 %). Vier Prozent der Befragten sind als Junior- oder Tenure-Track-Professor oder -Professorin beschäftigt. Ein weiterer Teil der Befragten hat andere Stellen inne, etwa eine wissenschaftliche Mitarbeit ohne Promotionsabsicht oder eine Nachwuchsgruppenleitung.

Etwa 71 % der Befragten geben an, bisher eine Karrierepause von weniger als 3 Monaten genommen zu haben. Bei etwa 11 % waren es 3 bis 6 Monate, bei ca. 9 % 7 bis 12 Monate und bei ca. 5 % 12 bis 24 Monate. Ihre Karriere für mehr als 24 Monate haben etwa 3 % der Befragten unterbrochen.

Die Befragten geben an, zu 50 % aus Haushaltsmitteln (z. B. Universität), zu 27 % aus Drittmitteln (z. B. DFG, Industrie), zu 2 % durch Stipendien und zu 21 % aus gemischten oder anderen Quellen finanziert zu sein. In den MINT-Fächern zeichnet sich ein 50/50-Verhältnis von Haushalts- zu Drittmitteln ab, in den Nicht-MINT-Fächern ein 75/25-Verhältnis. Gemischte oder andere Finanzierungsquellen scheinen zudem stärker bei Promovierenden eine Rolle zu spielen.

Der weit überwiegende Teil der Befragten (255 Personen) gibt an zu 100 % beschäftigt zu sein. In 50 %-Teilzeit arbeiten 46 Personen. Dazwischen wird noch von einigen Personen ein Beschäftigungsgrad von 70 % ausgewiesen (36 Personen). Ein Beschäftigungsgrad kleiner als 50 % wird nur von sehr wenigen der Befragten angegeben (13 Personen).

Das Arbeitsprofil unterschied sich zwischen beiden Gruppen nicht besonders. So lag der Median des Anteils der Lehrtätigkeit bei 20 % für MINT-Fächer und 30 % für Nicht-MINT-Fächer. Bezogen auf die Forschungstätigkeit ergab sich ein Median bei 50 % für MINT-Fächer und 40 % für Nicht-MINT-Fächer. Eine Auffälligkeit zeigte sich in der Anzahl der Personen, die für ihr Arbeitsprofil keinerlei Forschungstätigkeit angaben: 13 % in den Nicht-MINT-Fächern gegenüber nur 4 % in den MINT-Fächern.

Der derzeitig präferierte Karriereweg unterschied sich leicht zwischen den MINT- und Nicht-MINT-Fächern. Eine Führungskarriere in der Wirtschaft (21 % MINT und 16 % Nicht-MINT jeweils mit Präferenz 1 oder 2 von 5) oder eine fachliche Karriere in der Wirtschaft (36 % MINT und 29 % Nicht-MINT) wurde in beiden Gruppen von ähnlich vielen Personen angestrebt. Ebenso präferiert ein Großteil der Befragten in beiden Gruppen eine Karriere in der Wissenschaft mit Forschungsschwerpunkt (64 % MINT und 69 % Nicht-MINT). Unterschiede zeigten sich zwischen den Gruppen in Bezug auf eine Karriere in der Hochschullehre (39 % MINT und 67 % Nicht-MINT) und in der industrienahen Forschung (39 % MINT und 20 % Nicht-MINT).

## Herausforderungen

Die folgenden Fragen zielen auf die Bestandsaufnahme mehrerer Aspekte der von den Befragten erlebten Problematiken im Mittelbau. Es geht um typische Herausforderungen in den jeweiligen Qualifizierungsphasen sowie um Erfahrungen mit Stellenbewerbungen und Förderanträgen. Wir gehen auf Unterschiede bezüglich MINT und Nicht-MINT ein, falls diese auffallend bzw. erwähnenswert sind.

### Welche der nachfolgenden Herausforderungen erleben Sie persönlich in Ihrer aktuellen Qualifizierungsphase?

**Figure 2:**
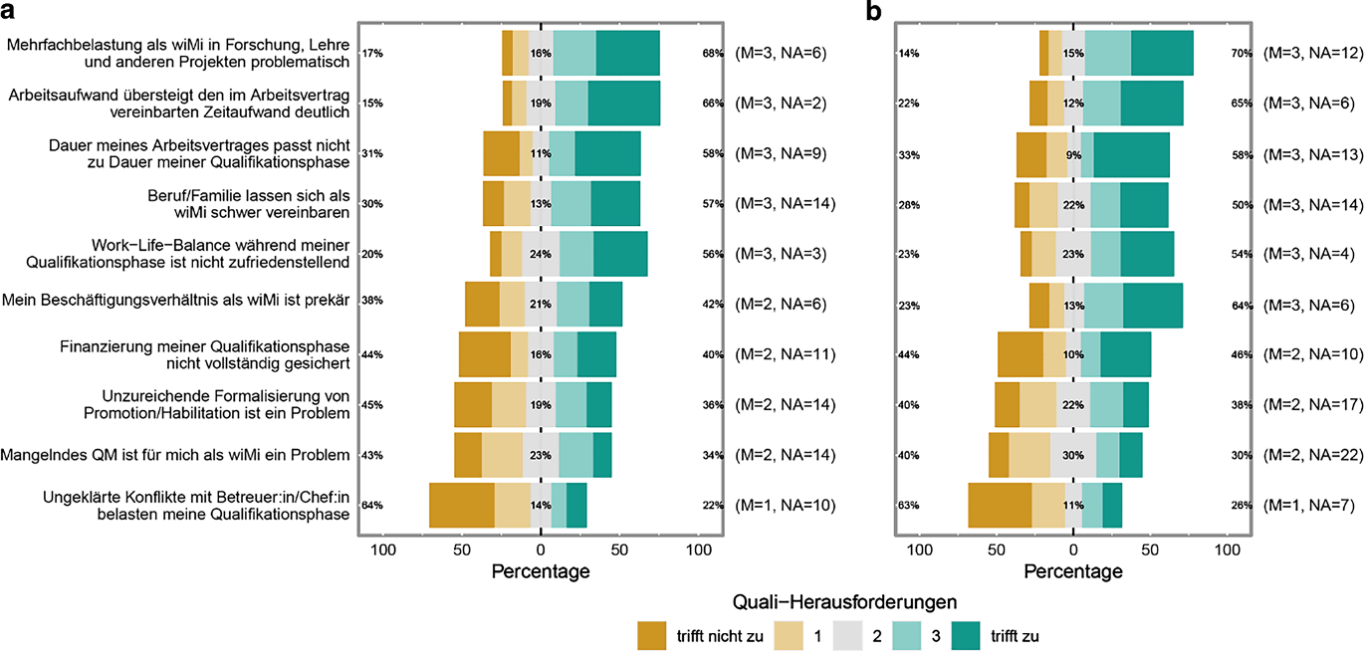

Hinsichtlich dieser Frage rangieren die Faktoren *Mehrfachbelastung (Forschung, Lehre etc.; 69* *%)* sowie *Überstunden* (66 %) an erster Stelle (Abb. 2). Im *MINT-Bereich* (Abb. 2a) wird das eigene *Arbeitsverhältnis oft als prekär* (42 %) empfunden, jedoch noch mehr im *Nicht-MINT-Bereich* (64 %, Abb. 2b). Die *mangelnde Finanzierung* gilt sowohl in den MINT- (40 %) als auch in den Nicht-MINT-Fächern (46 %) als eine vergleichbar starke Herausforderung. Auffallend ist, dass *Konflikte mit Betreuenden* fächerübergreifend als das geringste der gelisteten Probleme betrachtet wird (22 % bei MINT bzw. 26 % bei Nicht-MINT), was allerdings immer noch bedeutet, dass etwa jede und jeder vierte Befragte sich dieser Problematik ausgesetzt sieht.

### Wie oft haben sich die Befragten in ihrer wissenschaftlichen Laufbahn auf akademische Stellen beworben?

**Figure 3:**
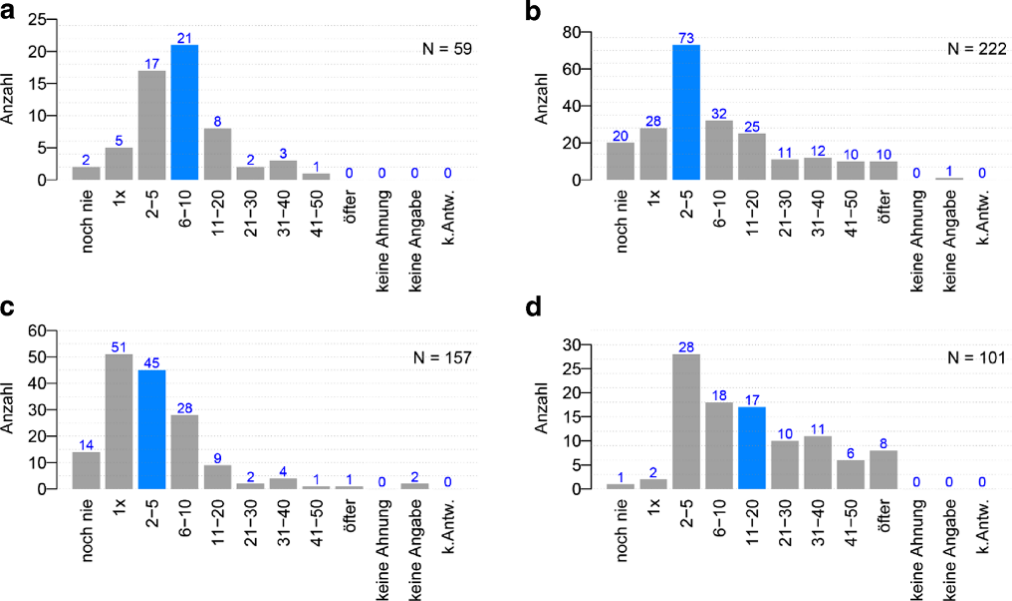

Über die beiden Fächergruppen hinweg liegt der Median bei 2–5 Bewerbungen (29 %, Abb. 3a bzw. 32 %, Abb. 3b), unabhängig von der Qualifikationsstufe. In den MINT-Fächern ist eine breitere Streuung als in den Nicht-MINT-Fächern zu verzeichnen, und zwar zwischen 1 und 10 Bewerbungen (Abb. 3a). In der Qualifikationsstufe *Postdoc oder Junior‑/Tenure-Track-Professoren und -Professorinnen* liegt die Anzahl der Bewerbungen generell um einiges höher – im MINT-Bereich liegt der Median bei 6–10 Bewerbungen (Abb. 3c), im Nicht-MINT-Bereich sogar bei 11–20 Bewerbungen, wobei die Bewerbungsanzahl jenseits der MINT-Fächer wesentlich breiter gestreut ist (Abb. 3d).

### Wie oft haben Sie in Ihrer wissenschaftlichen Laufbahn Forschungsförderanträge gestellt?

**Figure 4:**
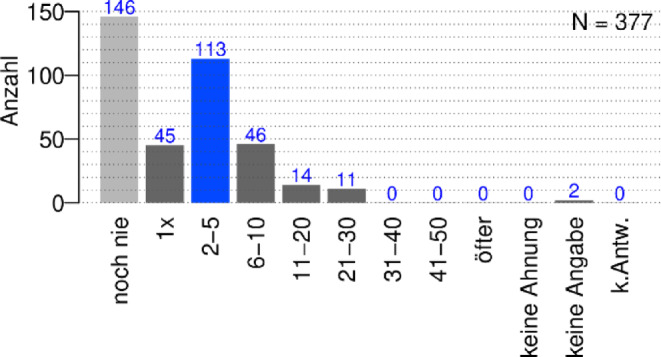

Unsere Daten zeigen ferner, dass etwa die Hälfte derjenigen, die während einer befristeten wissenschaftlichen Laufbahn im Mittelbau bereits Forschungsanträge gestellt haben, dies zwei- bis fünfmal tun (dunkelgraue und blaue Balken in Abb. 4). Etwa ein weiteres Viertel davon stellte 6–10 Anträge und das weitgehend unabhängig vom Fachgebiet. Circa 40 % der Befragten geben an, noch nie einen Antrag gestellt zu haben. Angenommen, eine durchschnittliche WiN-Karriere würde etwa 6 Jahre dauern und die durchschnittliche Förderzeit je Antrag etwa 2 Jahre, so wären im Schnitt etwa 3 Anträge zur Eigenfinanzierung dieser Zeit nötig. Man könnte daraus schließen, dass mehr als ein Drittel der Jungwissenschaftler und -wissenschaftlerinnen mehr als doppelt so viele Anträge schreiben als nötig. Da viele dieser Personen jedoch in dieser Zeit fremd-, lehr- und/oder nur teilfinanziert sind, ist diese Quote womöglich höher, was mit den einschlägigen Ablehnungsquoten übereinstimmen dürfte. Da das Schreiben von Anträgen sehr zeitaufwendig ist, bedeutet dies aber, dass dabei viel Zeit für die eigentliche Forschung verloren geht.

## Lösungsvorschläge

Die folgenden Fragen befassen sich mit der Analyse verschiedener Vorschläge für eine Verbesserung der Lage des wissenschaftlichen Nachwuchses. Wie im vorhergehenden Abschnitt gehen wir auch hier wieder auf Unterschiede bezüglich der beiden Bereiche MINT und Nicht-MINT ein.

### Welche der nachfolgenden Lösungsvorschläge halten Sie für zielführend für eine Verbesserung der Lage des wissenschaftlichen Mittelbaus?

**Figure 5:**
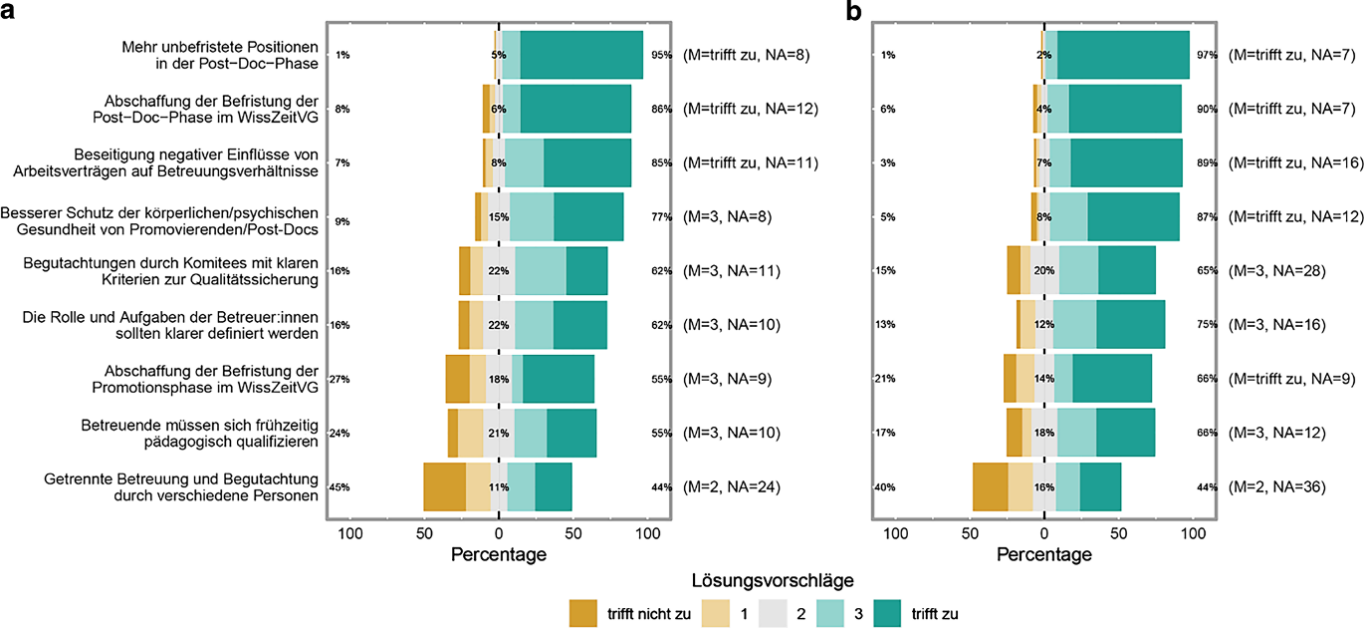

Der Vorschlag zur Ausweitung unbefristeter Positionen (d. h. Abschaffung des WissZeitVG) erfährt fachunabhängig unter Mitarbeitenden vor und nach der Promotion die größte Zustimmung (Abb. 5). Die Trennung von Begutachtung und Betreuung findet sich am unteren Ende der Reihenfolge (Abb. 5). Unabhängig von MINT und Nicht-MINT und Qualifikation ergibt sich hier ein vergleichsweise neutrales Bild, wobei sich Postdocs und Junior‑/Tenure-Track-Professor und -Professorinnen diese Trennung interessanterweise geringfügig stärker zu wünschen scheinen als Promovierende (ohne Abb.). Der einzig auffallende Unterschied in der Reihenfolge zwischen MINT und Nicht-MINT findet sich beim Vorschlag, *Begutachtungen durch Komitees auf Grundlage klarer Qualitätskriterien* durchzuführen (Abb. 5). In den MINT-Fächern deuten die Antworten dabei auf einen relativ gesehen stärkeren Verbesserungsbedarf hin als in den Nicht-MINT-Fächern.

### Welche weiteren Vorschläge halten Sie für zielführend? (Freitext)

Die Mehrzahl der Befragten spricht sich für *mehr unbefristete Stellen* nach der Promotion sowie alternative Karrierewege zur Professur aus. Mehr als ein Dutzend Mal wurde vorgeschlagen, Lehrstühle aufzulösen und eine *Departmentstruktur* einzurichten. Außerdem wird mehrfach angeregt, *Forschung und Lehre voneinander zu trennen *und mehr Personal, dass sich ausschließlich um die Lehre kümmert, unbefristet einzustellen. Des Weiteren sollte *Mehraufwand monetär entgolten *und die *Grundfinanzierung der Universitäten und Hochschulen aufgestockt werden*.

### Wen sehen Sie in der Verantwortung, die genannten Vorschläge umzusetzen?

**Figure 6:**
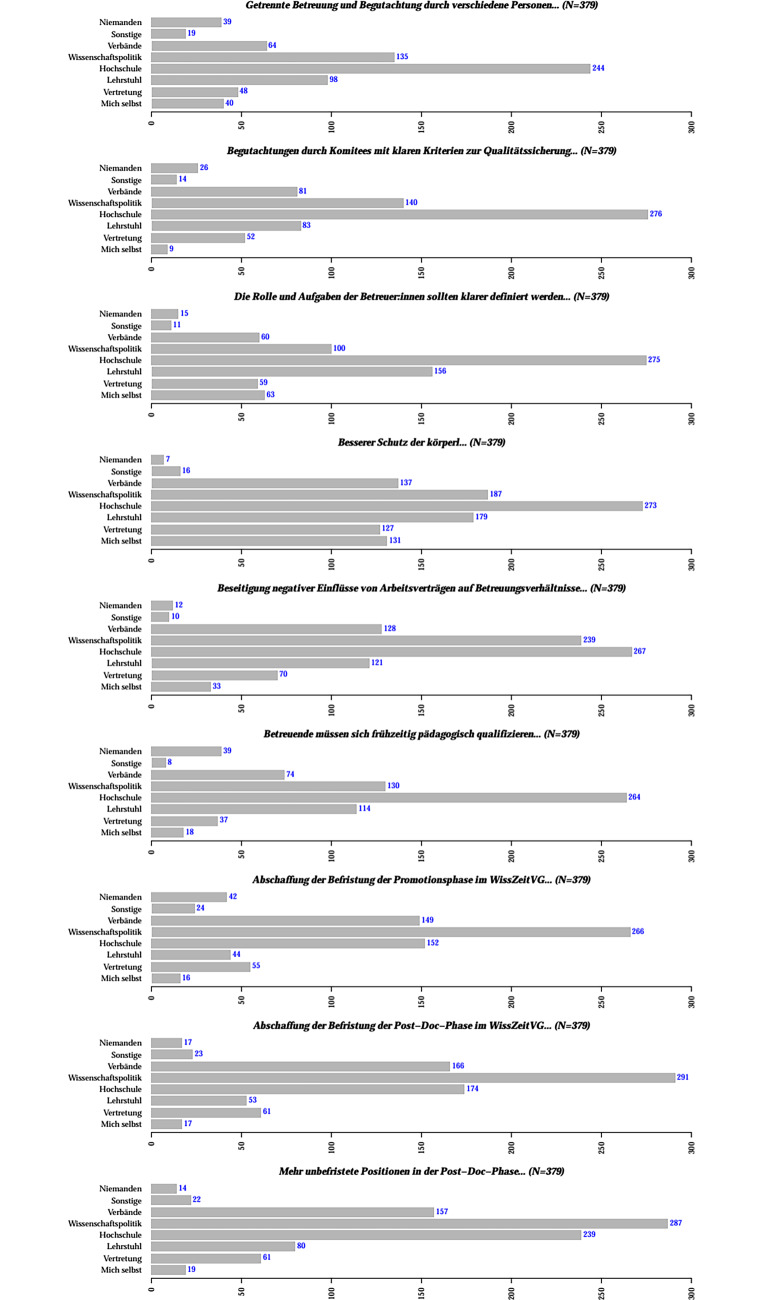

Interessanterweise decken sich bei dieser Mehrfachauswahl die Schwerpunkte fachbereichsübergreifend zwischen MINT und Nicht-MINT (Abb. 6). Wir fassen deshalb alle Antworten zusammen. Laut einer überwiegenden Mehrheit sollte die Problematik der *zeitlichen Befristung* von der Wissenschaftspolitik stärker in Angriff genommen werden. Zudem sieht eine überwiegende Mehrheit die Lösungen von Problemen in der *Betreuung von Qualifikationsphasen* und bzgl. der *Arbeitsbedingungen* eindeutig in der Verantwortung der Hochschulen. Hinsichtlich der *vertraglichen Rahmenbedingungen* sehen die meisten der Befragten die Wissenschaftspolitik und die Hochschulen gleichermaßen hauptverantwortlich für die Herbeiführung effektiver Lösungen.

### Wen würden Sie zur Bewältigung dieser Probleme als Ansprechpartner aufsuchen?

**Figure 7:**
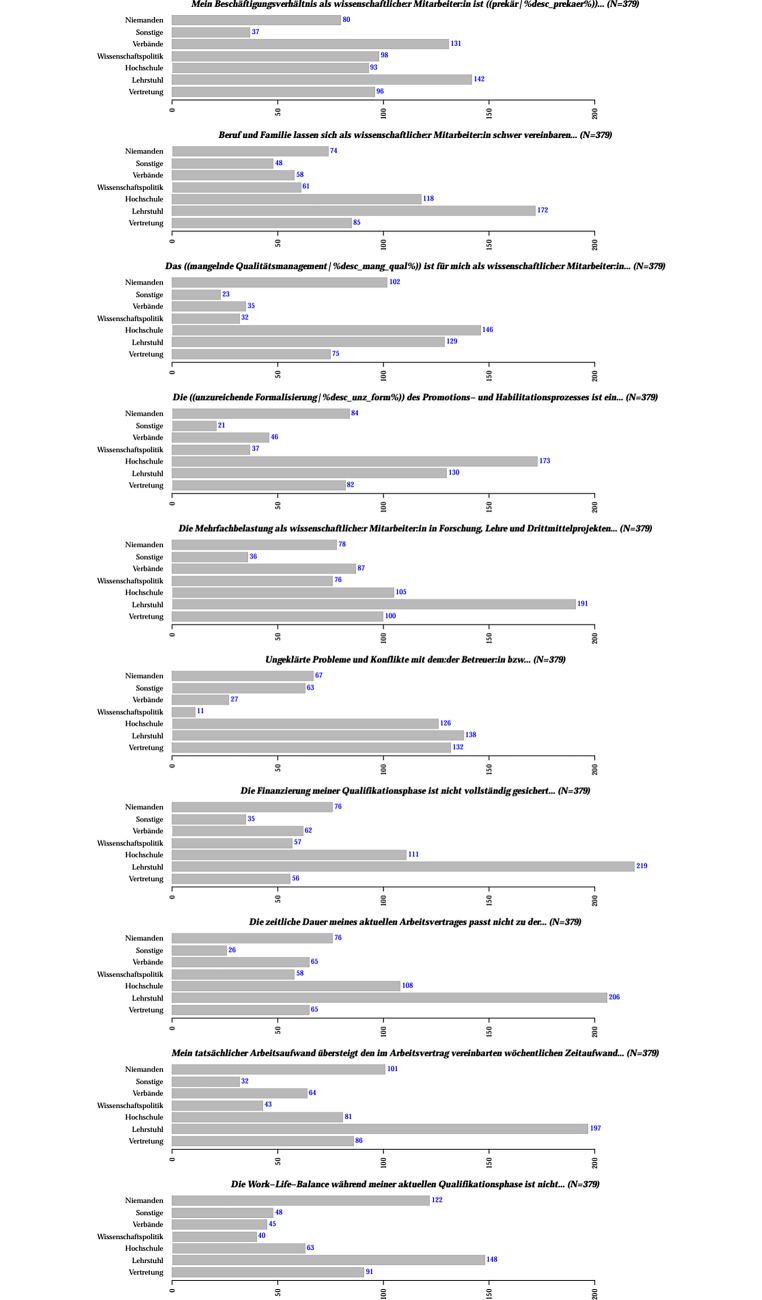

Hinsichtlich des mangelnden Qualitätsmanagements in der Qualifikationsphase sieht der überwiegende Teil der Befragten sowohl die Hochschule als auch den Lehrstuhl als erste Anlaufstelle (Abb. 7). Dies gestaltet sich ähnlich für die mangelnde Formalisierung des Promotionsprozesses – allerdings etwas stärker ausgeprägt für die Hochschulen als Ansprechpartner. Besonders auffällig ist, dass die Befragten bei den Fragen zur Finanzierung und der zeitlichen Dauer der Qualifikationsphase ihren Lehrstuhl als Kontaktpartner betrachten. Dies gilt auch für Fragen zu dem tatsächlich geleisteten Arbeitsaufwand und der Work-Life-Balance allgemein.

### Für wie sinnvoll und unterstützenswert halten Sie folgende Lösungsansätze für eine erfolgreiche Doktorandenbetreuung?

**Figure 8:**
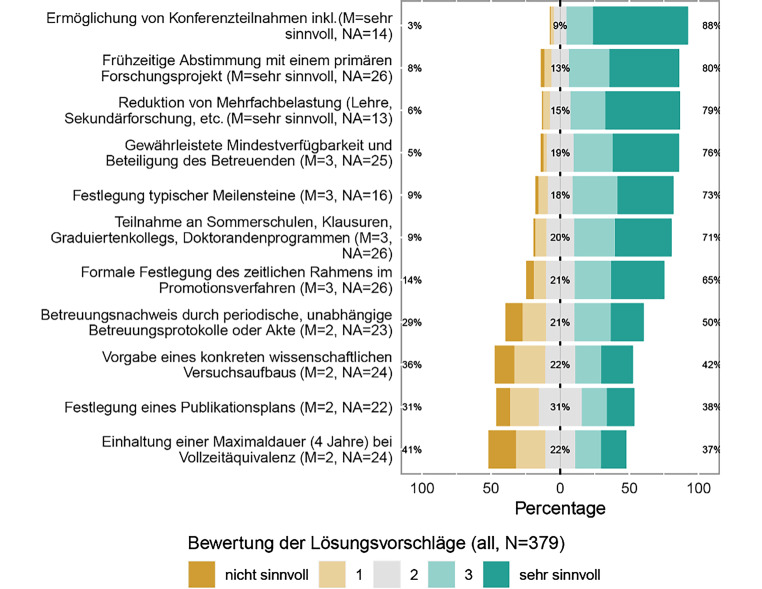

Auch hier zeigt sich zwischen MINT und Nicht-MINT hinweg eine homogene Bewertung der Lösungsvorschläge (Abb. 8). Überwiegend als sinnvoll oder sehr sinnvoll werden Maßnahmen bewertet, die eine aktive Beteiligung an der Wissenschaftsgemeinschaft ermöglichen. Hier werden sowohl *Konferenzteilnahmen mit eigenen Beiträgen* als auch die *Teilnahme an internen Veranstaltungen* wie etwa Lab Retreats als sinnvoll erachtet. Auch Maßnahmen, die eine stärkere Fokussierung auf Forschungstätigkeit in der Promotionsphase erlauben, werden sehr positiv aufgenommen. Hier sind die *Abstimmung mit einem primären Forschungsprojekt* und die *Begrenzung von Zusatzaufgaben*, beispielsweise Lehrverpflichtungen, zu nennen.

Weitere als überwiegend sinnvoll erachtete Maßnahmen lassen sich unter einer strukturierten und aktiven Betreuungssituation zusammenfassen. Hierunter fallen sowohl die *Festlegung typischer Meilensteine* und die *Festlegung eines formalen zeitlichen Rahmens*, etwa bezogen auf Bewertungszeiträume, als auch die *Gewährleistung einer Mindestverfügbarkeit und -beteiligung der oder des Betreuenden*. Ein *Nachweis dieser Betreuung* etwa in Form einer Betreuungsakte wird zwar immer noch mehrheitlich, aber schon deutlich seltener als sinnvoll eingeschätzt.

Die Sinnhaftigkeit anderer Maßnahmen wird deutlicher diverser bewertet. Die *Festlegung eines Publikationsplans*, einer *Maximaldauer der Promotionsphase* und auch die *Vorgabe eines konkreten wissenschaftlichen Umfelds*, beispielsweise in Form einer Forschungsmethode, werden von etwa gleich vielen Befragten als sinnvoll beziehungsweise nicht sinnvoll eingestuft.

### Welche weiteren Maßnahmen sehen Sie als zielführend für eine erfolgreiche Doktorandenbetreuung an? (Freitext)

In jedem fünften Vorschlag werden Maßnahmen zu einem besseren persönlichen und fachlichen Austausch, etwa in Form von Mentoring, Peer-Gruppen oder auch Auslandssemestern, erwähnt. Weitere Vorschläge beziehen sich auf das Betreuungsverhältnis. Hier werden Maßnahmen zur Qualifizierung der oder des Betreuenden, regelmäßige Feedbackgespräche, geteilte Betreuungen durch mehrere Personen und auch eine Begrenzung der Anzahl der Betreuungsverhältnisse genannt. Zusätzlich werden Vorschläge zur vertraglichen Gestaltung der Promotion als Qualifikationsmaßnahme gemacht. Insbesondere die Festlegung eines Arbeitszeitanteils für die ausschließliche Arbeit an der Promotion und auch die praktische Ermöglichung dieser Arbeit werden gefordert.

Stellvertretend sind hier 2 Beiträge zitiert, die eine stärkere Formalisierung des Promotionsprozesses kritisch sehen:

In den Geisteswissenschaften kann eine zu enge Formalisierung klar die Forschung negativ beeinflussen.

Bei allen Wünschen für mehr Struktur und Vorgaben: Es braucht Flexibilität, wenn im Laufe der Jahre Dinge sich nicht so entwickeln, wie ursprünglich geplant.

## Einfluss der COVID-19-Pandemie

Die folgenden Fragen zielen auf den Einfluss der derzeitigen COVID-19-Pandemie auf die Lage des wissenschaftlichen Nachwuchses ab. Da die Antworten sich kaum für die Bereiche MINT und Nicht-MINT unterscheiden, stellen wir lediglich die Gesamtergebnisse dar.

**Figure 9:**
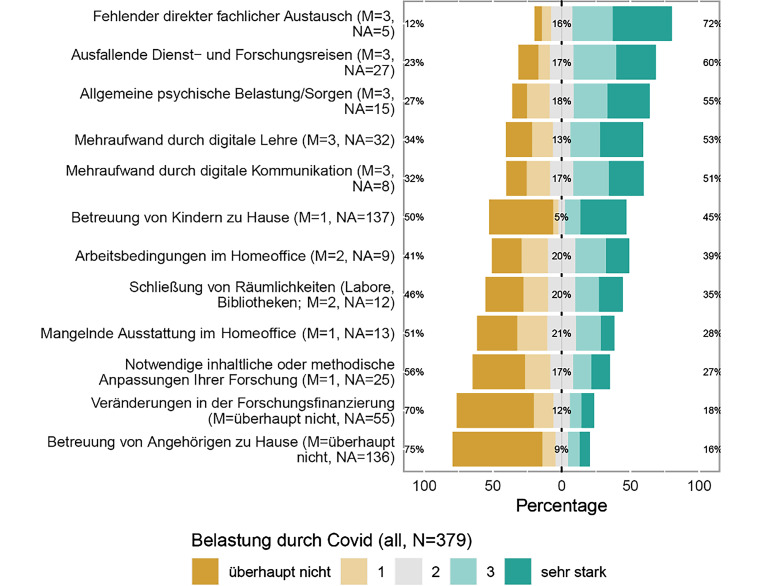

Als größte Belastung im Rahmen der Coronapandemie kann der fehlende direkte fachliche Austausch identifiziert werden (72 %; Abb. 9). Darüber hinaus, aber mit einigen Prozentpunkten Abstand, werden ausfallende Dienst- und Forschungsreisen (60 %), die allgemeine psychische Belastung (55 %) und der durch digitale Lehre induzierte Mehraufwand (53 %) als belastend empfunden.

Als weniger belastend scheinen anhand der Datenlage die notwendigen inhaltlichen und methodischen Anpassungen der Forschung (27 %), Veränderungen in der Forschungsfinanzierung (18 %) und die Betreuung von Angehörigen zu Hause (16 %) wahrgenommen zu werden.

**Figure 10:**
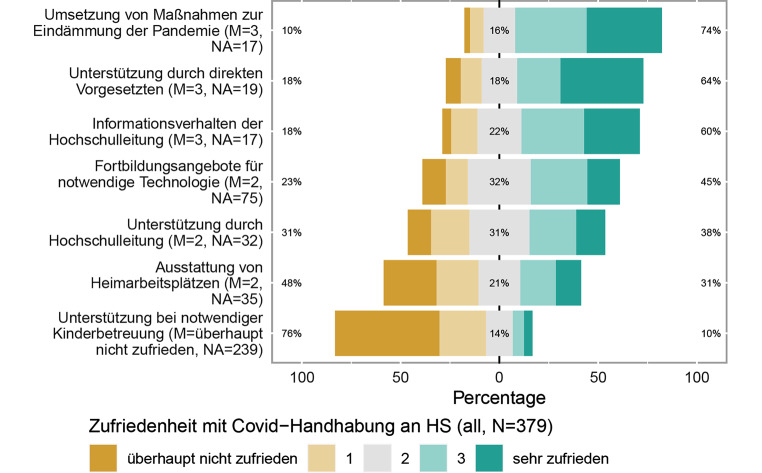

Der überwiegende Anteil der Befragten ist zufrieden mit der Umsetzung eindämmender Maßnahmen zur Pandemie (75 %; Abb. 10). Auch die Unterstützung durch direkte Vorgesetzte (64 %) und das Informationsverhalten der Hochschulleitung (60 %) werden überwiegend positiv wahrgenommen. Dagegen stoßen die Ausstattung von Heimarbeitsplätzen (48 %) und vor allem die Unterstützung bei notwendiger Kinderbetreuung (76 %) eher auf Unzufriedenheit.

## Diskussion und Fazit

Im folgenden Abschnitt wollen wir unsere erzielten und dargestellten Ergebnisse auf den derzeitigen Stand des öffentlichen Diskurses beziehen. Hierbei gehen wir anhand von verschiedenen relevanten Problematiken vor, die sich aus der Umfrage ergeben haben.

### Prekäre Beschäftigungsverhältnisse

Der wissenschaftliche Nachwuchs ist prekären Beschäftigungsverhältnissen unterworfen. So existiert entlang der akademischen Karriereleiter ein unsichtbarer Flaschenhals auf dem Weg zur Professur [1], welche oftmals die einzige Möglichkeit für eine unbefristete Stelle im deutschen Hochschulbetrieb darstellt [3]. Das 2007 eingeführte WissZeitVG, das ursprünglich dazu angedacht war, Promovierende schnell in sichere Arbeitsverhältnisse zu überführen, verschärfte diese Lage: Permanente Stellen an Hochschulen und Universitäten wurden massiv abgebaut und die Vertragslaufzeiten wurden immer kürzer [1, 4, 5].

So stellt auch das Netzwerk für gute Arbeit in der Wissenschaft (NGAWiss) fest, dass über 80 % der Personen im wissenschaftlichen Mittelbau an deutschen Hochschulen befristet angestellt sind, obwohl sie einen Großteil der Forschung und Lehre tragen. Eine Aussicht auf Verbleib in der Wissenschaft haben sie nicht [3]: Der Anteil an unbefristeten Stellen im wissenschaftlichen Mittelbau liegt bei ca. 10 % [2, 6]. Im Bundesbericht für den wissenschaftlichen Nachwuchs wird zudem festgehalten, dass etwa 10 Jahre nach einer Promotion lediglich jede fünfte promovierte Person noch an Hochschulen oder außeruniversitären Forschungseinrichtungen beschäftigt ist [2].

Entsprechend nachvollziehbar ist es, dass sowohl die Entfristung weiterer Stellen für Post-Doktoranden und -Doktorandinnen als auch die Abschaffung des WissZeitVGs (95 %) eine sehr hohe Zustimmung im Rahmen unserer Umfrage haben (86 %).

### Machtmissbrauch in der Wissenschaft

Dreh- und Angelpunkt in der akademischen Laufbahn ist die Professur [3]. Unmittelbar damit verknüpft sind verschiedene Abhängigkeitsverhältnisse von Promovierenden und Postdocs zur Professorin bzw. zum Professor, die sich oftmals in steilen Hierarchien widerspiegeln. So ist die Professorin beziehungsweise der Professor in der Regel Führungskraft, Betreuer/Betreuerin und Erstgutachter/Erstgutachterin der wissenschaftlichen Mitarbeitenden und Promovierenden. Aufgrund dieser dreigliedrigen Machtposition finden sich wissenschaftliche Mitarbeitende in einem starken Abhängigkeitsverhältnis im deutschen Wissenschaftssystem wieder [7].

Erschwerend hinzukommen fehlende Trainings in der Personalführung wissenschaftlicher Führungspersonen und das Fehlen zuverlässiger und vertrauenswürdiger Mechanismen, Konflikte zu melden und zu lösen [7, 8]. Dies resultiert in gravierenden Folgen, etwa einer fehlenden Diskussionskultur und Vollzeitbelastung auf Teilzeitstellen, was den Leitlinien einer guten wissenschaftlichen Praxis diametral gegenübersteht [9].

Diese Problematik spiegelt sich entlang der Ergebnisse des Fragebogens auch wider, allerdings relativ zu anderen Herausforderungen weniger stark ausgeprägt. So betrachten immerhin 24 % der Befragten Konflikte mit ihrem Betreuenden als eine Herausforderung in ihrer Promotionsphase. Deutlich stärker repräsentiert ist dagegen die Ansicht, dass die Begutachtung ihrer Qualifikationsarbeit von einem Komitee auf Basis klar definierter Kriterien durchgeführt werden sollte (62 % Zustimmung bei MINT, 65 % bei Nicht-MINT).

### Arbeitsbelastung und Mehrarbeit

Ein weiterer Aspekt, mit dem sich der wissenschaftliche Mittelbau konfrontiert sieht, ist die hohe Arbeitsbelastung. So existiert eine deutliche Diskrepanz zwischen den vertraglich vereinbarten Stunden und den tatsächlich geleisteten Arbeitsstunden von wissenschaftlichen Mitarbeitenden. Seipel et al. [10] haben diese beispielsweise für die Universität Hildesheim erhoben. Sie stellen heraus, dass bei Vollzeitstellen die tatsächlich geleistete Arbeitszeit zumindest zusätzliche 8,6 h wöchentlich umfasst. Noch extremer ist dieser Unterschied bei 50-Prozent-Stellen, im Rahmen derer wöchentlich 15,5 h zusätzlich geleistet werden und damit nahezu doppelt so viele Arbeitsstunden wie vertraglich vereinbart. Effektiv reichen diese dadurch fast an die Vertragszeit von Vollzeitstellen heran [10]. Dabei explizieren die Autoren nicht, welche finanziellen Einbußen diese Praxis auf die Gehälter von Wissenschaftlern und Wissenschaftlerinnen hat.

Die Gewerkschaft Erziehung und Wissenschaft mahnt an, dass der wissenschaftliche Mittelbau 40.000 zusätzliche Stellen benötigt. An Hochschulen für angewandte Wissenschaften sollten zusätzliche 10.000 Stellen erstellt werden, um der Aufgabenstellung aus Forschung, Lehre, Nachwuchsförderung und Wissenstransfer gerecht zu werden [4]. An Universitäten kommen aktuell 60 Studierende auf gerade einmal eine Professorin oder einen Professor [5], wodurch auch die Qualität ihres Studiums leidet.

Laut dem Deutschen Zentrum für Hochschul- und Wissensforschung (DZHW) ist die geleistete Mehrarbeit unter Wissenschaftlern und Wissenschaftlerinnen unabhängig von der Befristung ihres Arbeitsvertrags: Sowohl befristete als auch unbefristete Wissenschaftler und Wissenschaftlerinnen arbeiten mehr als vertraglich vereinbart [11].

Die Mehrfachbelastung wurde im Zuge des Fragebogens auch als die größte Herausforderung wahrgenommen (69 %). Dies hat sich in der COVID-19-Pandemie noch verschärft.

### Chancengleichheit und Gerechtigkeit sowie unzureichende Vereinbarkeit von Beruf und Familie

Die Vereinbarkeit von Beruf und Familie gestaltet sich in einem Berufsverhältnis im wissenschaftlichen Mittelbau als schwierig. Dabei führen die unsicheren Beschäftigungsbedingungen insbesondere zu einer Benachteiligung von Wissenschaftlerinnen [1, 2]. Es ist anzunehmen, dass bei Wissenschaftlerinnen aufgrund mangelnder beruflicher und finanzieller Sicherheit ein hoher Anteil kinderlos bleibt [2]. Knapp 40 % der wissenschaftlich und künstlerisch Beschäftigten der Universität Hildesheim [10] und Universität Trier [12] stellen ihre Kinderwünsche aufgrund von beruflichen Gründen zurück. Dies deckt sich mit den Ergebnissen des Bundesberichts für den Wissenschaftlichen Nachwuchs: Etwa ein Sechstel der Promovierenden und die Hälfte der Promovierten an Hochschulen sind Eltern. Zudem gründen diese seltener eine Familie als altersgleiche Hochschulabsolventinnen und -absolventen, die ein Beschäftigungsverhältnis außerhalb der Wissenschaft haben [2].

Besonders auffällig ist zudem die Ungleichverteilung zwischen Professorinnen und Professoren [13, 14]: Es gibt zwar Fächer, in denen die Hälfte der Promovierenden weiblich ist, jedoch diese Quote bei der Verteilung von Professoren und Professorinnen noch nicht einmal 20 % erreicht [13]. Um den Frauenanteil zu erhöhen beziehungsweise um auch für Männer mehr Sicherheit in einem wissenschaftlichen Arbeitsverhältnis zu schaffen, ist der Aufbau von Tenure-Track-Stellen eine wünschenswerte Möglichkeit [13].

Die schwierige Vereinbarkeit von Beruf und Familie in einer Tätigkeit im wissenschaftlichen Apparat deckt sich auch mit den Umfrageergebnissen in Bezug zu den Maßnahmen während der Coronapandemie. Etwa 76 % der Befragten sind überhaupt nicht zufrieden mit der Unterstützung ihrer Hochschule bei der Kinderbetreuung. Zudem halten 55 % der Befragten die Work-Life-Balance für nicht zufriedenstellend.

### Mangelndes Qualitätsmanagement und fehlende Stellen am Arbeitsmarkt

Eine weitere Herausforderung des wissenschaftlichen Mittelbaus stellt ein mangelndes Qualitätsmanagement der Betreuung und Führung dar [1]. Dies spiegelt sich in fehlenden Mechanismen zur Konfliktbewältigung, einem hohen Publikationsdruck und ungeschulten Führungspersönlichkeiten wider [1].

Auch die unzureichende Formalisierung der Prozesse im wissenschaftlichen Apparat trägt zu einer mangelnden Qualitätssicherung bei. So sind Bewerbungs- und Betreuungsprozesse im Rahmen einer Promotion oder auf dem Weg zur Habilitation nicht ausreichend schriftlich fixiert [1]. Es besteht ein breites Spektrum hinsichtlich der Formalisierung von Promotionsmodellen [15]. Auf Empfehlung der Salzburg Principles soll die Formalisierung der Promotionsphase allerdings gerade durch schriftlich fixierte Vereinbarungen geregelt werden [2]. Auch dies ist anhand unserer Datenlage erkennbar; so erhält der Lösungsvorschlag, die Aufgaben von Betreuenden zu explizieren, zum Beispiel durch Festlegung von Meilensteinen, des Themas und des zeitlichen Rahmens, viel Zustimmung seitens der Befragten.

Das Tenure-Track-Verfahren, das bisweilen den Weg in eine Professur bereiten soll, ist dagegen ausreichend formuliert. Allerdings ist dieses nominell nur ein Anfang. So stehen den 1000 zusätzlichen Stellen, die der Bund über den Tenure-Track schaffen möchte, ca. 160.000 Qualifizierte gegenüber [16]. Laut der Gemeinsamen Wissenschaftskonferenz [17] gab es 2018 71.193 Bewerbungen auf 3059 ausgeschriebene Professuren. Die Gewerkschaft für Erziehung und Wissenschaft fordert u. a. deshalb zusätzliche 5000 Tenure-Track-Professuren sowie 40.000 Dauerstellen an den Universitäten [2]. Juniorprofessuren ohne Tenure-Track-Option haben oft keine langfristige Perspektive. Ihre Stellen sind in der Regel auf 6 Jahre befristet, wodurch sie sich abermals mit einer beruflichen Planungsunsicherheit konfrontiert sehen [18].

### Hochschulorganisation

An deutschen Universitäten fehlen selbstständig tätige Hochschullehrer unterhalb der Professur fast ganz [19]. Dreh- und Angelpunkt in der akademischen Laufbahn in Deutschland ist die Professur [3]. Allerdings existiert eine anhaltende Debatte darüber, die bestehenden Personalstrukturen, das heißt Lehrstühle, im deutschen Wissenschaftssystem in z. B. Departmentstrukturen zu überführen (u. a. [3, 20]). Damit verbindet die Junge Akademie eine weitgehende „Umwandlung von Qualifikationsstellen in Professuren“ [20]. Dieser Missstand ist in unserer gesammelten Datenlage eindeutig erkennbar. So gibt es eine sehr deutliche Zustimmung, dass Postdoc-Stellen entfristet werden sollen (97 %).

## Zusammenfassung

In diesem Artikel haben wir die Ergebnisse unserer Umfrage unter Angehörigen des wissenschaftlichen Nachwuchses dargestellt. Es zeigte sich, dass diese vielfältigen Herausforderungen begegnen müssen, die in Nicht-MINT-Fächern und MINT-Fächern ähnlich empfunden werden. Lediglich die Verfügbarkeit von Stellen könnte sich basierend auf der Anzahl an Bewerbungen im MINT-Bereich etwas besser darstellen als im Nicht-MINT-Bereich. Auch bei der Bewertung von Lösungsvorschlägen zeigte sich ein homogenes Bild über die Gruppen. Insbesondere die Schaffung von unbefristeten Stellen für die Postdoc-Phase wurde hier von nahezu allen Befragten gefordert. Die letzten zwei Jahre im Zeichen der COVID-19-Pandemie waren für einen großen Teil der Befragten von fehlendem fachlichen Austausch und weiteren Belastungen geprägt. Insgesamt leistet die Umfrage einen Beitrag dazu, die Probleme des wissenschaftlichen Nachwuchses und notwendige Verbesserungsmaßnahmen zu verdeutlichen.
